# Dual Modulation of Infection and Skin Recovery by Lamiaceae Hydrolate Hydrogels in *S. aureus*-Infected Burns

**DOI:** 10.3390/antibiotics15010020

**Published:** 2025-12-22

**Authors:** Grigory Demyashkin, Mikhail Parshenkov, Alibek Tokov, Tatiana Sataieva, Anatoly Kubyshkin, Vladimir Shchekin, Sergey Popov, Boris Kuzminov, Nadezhda Zabroda, Artem Volodkin, Kirill Blinov, Petr Shegay, Andrei Kaprin

**Affiliations:** 1 Department of Digital Oncomorphology, National Medical Research Centre of Radiology, 2nd Botkinsky Pass, 3, 125284 Moscow, Russia; misjakj@gmail.com (M.P.); dr.shchekin@mail.ru (V.S.); dr.shegai@mail.ru (P.S.); kaprin@mail.ru (A.K.); 2 Laboratory of Histology and Immunohistochemistry, Institute of Translational Medicine and Biotechnology, I.M. Sechenov First Moscow State Medical University (Sechenov University), Trubetskaya Str, 8/2, 119048 Moscow, Russia; zabroda_n_n@staff.sechenov.ru (N.Z.); pyrk2@yandex.ru (K.B.); 3 Research and Educational Resource Center for Immunophenotyping, Digital Spatial Profiling and Ultrastructural Analysis Innovative Technologies, Peoples’ Friendship University of Russia (RUDN University), Miklukho-Maklaya Str, 6, 117198 Moscow, Russia; tokov.alibek@bk.ru (A.T.); volodkin-av@rudn.ru (A.V.); 4 Department of Microbiology, Virology and Immunology, Medical Institute Named After S.I. Georgievsky, Crimean Federal University Named After V.I. Vernadsky, Lenina Boulevard, 5/7, 29505 Simferopol, Russia; tanzcool@mail.ru (T.S.); boris_kuzminov@mail.ru (B.K.); 5 General and Clinical Pathophysiology Department, Medical Institute Named After S.I. Georgievsky, Crimean Federal University Named After V.I. Vernadsky, Lenina Boulevard, 5/7, 29505 Simferopol, Russia; anatoly2802@gmail.com; 6 Department of General Practice, Medical Institute, Peoples’ Friendship University of Russia (RUDN University), Miklukho-Maklaya Str 6, 117198 Moscow, Russia; popov_serv@pfur.ru; 7 Department of Urology and Operative Nephrology, Peoples’ Friendship University of Russia (RUDN University), Miklukho-Maklaya Str 6, 117198 Moscow, Russia

**Keywords:** burn wound, hydrogel, *Staphylococcus aureus*, *Satureja montana*, *Origanum vulgare*, plant extracts, rabbit model, Ki-67, wound healing

## Abstract

**Background/Objectives:** Burn wound infections caused by *Staphylococcus aureus* remain a major clinical challenge, leading to delayed healing and high mortality. Natural compounds derived from the *Lamiaceae* family possess antimicrobial and anti-inflammatory properties that may modulate wound recovery. This study aimed to evaluate the dual modulatory effects of *Satureja montana* and *Origanum vulgare* hydrolate-loaded hydrogels on modulation of infection and skin recovery in an experimental rabbit model of *S. aureus*-infected burns. **Methods:** Full-thickness (grade IIIa) thermal burns were induced in 25 male New Zealand White rabbits, followed by inoculation with *S. aureus* (10^8^–10^9^ CFU/mL). Animals were divided into five groups: sham control, burn-infection control, standard-of-care intervention, *Satureja montana* hydrolate intervention, and *Origanum vulgare* hydrolate intervention. Treatments were applied twice daily for 14 days. Bacterial load (CFU/g), biochemical markers, histological parameters, and multiplex immunohistochemical indices (Ki-67, CD68, CD163) were analyzed. **Results:** Both hydrolate-based formulations exhibited pronounced antibacterial effects, significantly reducing *S. aureus* counts by day 14 compared to untreated burns (*p* < 0.001). Immunohistochemical analysis revealed enhanced cell proliferation and a rapid shift from pro-inflammatory M1 (CD68+) to reparative M2 (CD163+) macrophages, indicating effective immune resolution. The hydrolate-loaded hydrogels effectively combined antimicrobial activity with tissue-regenerative and immunomodulatory effects. The *S. montana* formulation demonstrated superior performance, representing a promising adjunctive therapy for infected burn wounds. **Conclusions:** This study represents the first comparative in vivo evaluation of *S. montana* and *O. vulgare* hydrolate-loaded hydrogels in a complex *S. aureus*-infected burn model.

## 1. Introduction

Thermal trauma (or burn injury) stands as a pervasive and devastating form of physical insult, consistently ranking among the leading causes of global morbidity and mortality [1,2]. The global burden of these injuries is substantial, with recent estimates indicating that between 7 and 12 million people sustain burns annually, resulting in approximately 300,000 associated fatalities per year [3,4,5]. This profound impact is further underscored by the high mortality rates, which can reach up to 25% in severe cases [6]. Even in developed healthcare systems, such as the United States, an estimated 600,000 burn injuries annually necessitate emergent medical intervention, highlighting the persistent and significant public health challenge [7]. Despite the variability in reporting methodologies, the overarching conclusion remains unequivocal: burn injuries impose a protracted burden of morbidity and mortality, making the development of effective therapeutic strategies a critical priority.

Critically, infection emerges as the predominant driver of adverse outcomes in hospitalized burn patients, leading to prolonged hospital stays and complicating reconstructive efforts [8]. Following thermal trauma, the skin’s protective barrier is breached, exposing a nutrient-rich, moist, and warm environment highly conducive to microbial proliferation and biofilm formation [9]. This creates an urgent clinical challenge, as bacterial infections are implicated in up to 75% of burn-related fatalities [10].

While a diverse array of opportunistic pathogens can colonize burn wounds, the scientific literature has extensively focused on Gram-negative bacteria: *Pseudomonas aeruginosa* and *Klebsiella pneumoniae*. Numerous preclinical and clinical studies have elucidated their pathogenic mechanisms and explored therapeutic strategies, including bacteriophage therapy, with some success in animal models [11,12,13]. These investigations have significantly advanced our understanding of burn wound infections caused by these specific organisms.

However, a critical lacuna exists in the research landscape concerning *Staphylococcus aureus* (*S. aureus*), despite its consistent identification as a predominant early invader and major causative agent in clinical burn units [14]. This Gram-positive bacterium poses unique and formidable challenges due to its inherent capacity for robust biofilm formation and the alarming rise in antibiotic-resistant strains, particularly methicillin-resistant *S. aureus* (MRSA) [15]. The virulence of *S. aureus* is further amplified by its secretion of potent toxins, which can trigger an exaggerated pro-inflammatory response, leading to a spectrum of complications from superficial impetigo to severe invasive infections and sepsis [16]. Biofilms, acting as protective matrices, render *S. aureus* highly recalcitrant to both host immune defenses and conventional antimicrobial agents, thereby perpetuating infection and complicating treatment [17]. The progression to invasive infection, characterized by high bacterial loads (>10^5^ CFU/g of tissue) and tissue necrosis, constitutes a surgical emergency with substantial risk of systemic complications and mortality [9]. Furthermore, the complex, biphasic immune response characteristic of severe burns often confounds the diagnosis of sepsis, necessitating specialized diagnostic criteria for this patient population [18]. Despite these well-recognized clinical implications and the significant threat *S. aureus* poses, the volume of robust clinical and translational research specifically addressing *S. aureus* in burn wound infections remains disproportionately low compared to other prevalent pathogens, particularly regarding novel therapeutic interventions and in vivo models. This highlights an urgent need for targeted research to develop effective strategies against *S. aureus* in the challenging microenvironment of burn wounds.

Natural products, especially those derived from medicinal plants, offer a rich reservoir of compounds with potential antimicrobial and anti-inflammatory properties. The *Lamiaceae* family, renowned for its aromatic species, has garnered significant scientific interest for its diverse phytochemical profile and established biological activities [19]. Among these, *Origanum vulgare* (oregano) and *Satureja montana* (mountain savory) are particularly notable for their potent antimicrobial efficacy, largely attributed to phenolic compounds such as carvacrol and thymol [20]. These compounds exert their effects through various mechanisms, including disruption of bacterial cell membranes, inhibition of enzyme activity, and interference with efflux pumps, thereby offering a multi-targeted approach against pathogens like *S. aureus* [21,22].

Hydrolates, the aqueous co-products of essential oil distillation, represent a sustainable and often less cytotoxic alternative to concentrated essential oils, retaining water-soluble aromatic compounds and exhibiting antimicrobial properties [23]. The application of these plant-derived agents within hydrogel delivery systems presents a compelling strategy for burn wound management. Hydrogels, as biocompatible and moisture-retaining matrices, provide an ideal environment for wound healing, facilitate sustained release of therapeutic agents, and can physically protect the wound bed [24].

The integration of *Lamiaceae* hydrolates into hydrogels thus offers a dual-action potential: directly combating microbial infection, particularly from resistant strains like *S. aureus*, and modulating the inflammatory response inherent to burn injuries. While in vitro studies have demonstrated the antimicrobial efficacy of *O. vulgare* and *S. montana* hydrolates against various pathogens, including *S. aureus*, and early in vivo models have shown promising results in burn wound healing, there remains a significant research gap concerning their long-term efficacy and impact in more complex, delayed infection models [25,26].

Addressing this gap is crucial for translating the therapeutic potential of *Lamiaceae* hydrolate-loaded hydrogels into effective clinical applications for *S. aureus*-infected burns.

Based on this critical need, the primary aim of the present study was to investigate the dual modulatory effects of *Lamiaceae* hydrolate-loaded hydrogels on infection control and tissue repair in a complex *Staphylococcus aureus*-infected burn wound model.

To achieve this aim, the following specific objectives were established:

1. To conduct a bacteriological analysis of skin tissue samples to assess microbial contamination and determine the antibacterial effects of gels based on Lamiaceae hydrolate-loaded hydrogels.
2. To perform histological analysis of skin tissues for morphological evaluation of regenerative processes and assessment of tissue damage following the application of the hydrolate-based gels.
3. To perform multiplex immunohistochemical analysis of Ki-67, M1, and M2 macrophage markers to evaluate cellular proliferation and the dynamic balance of inflammatory and reparative macrophage phenotypes within burn wounds.

## 2. Material and Methods

### 2.1. Experimental Animals

New Zealand White rabbits (male, 8 weeks old; 3.0 ± 0.35 kg) were acclimatized for seven days before the experiment. Animals were individually housed in stainless steel cages (4 square feet of floor space and a height of 20 inches to allow for natural posture and comfort) with wire mesh floors, allowing social contact. Cages were enriched with rice husk bedding (Delta Feeds, Moscow, Russia) and gnawing blocks. The vivarium maintained a controlled environment: 21 ± 1 °C, 40–60% humidity, and a 12L:12D cycle (lights on 07:00–19:00). Rabbits received access to standard laboratory chow (Delta Feeds, Moscow, Russia) and purified water via automated systems (ad libitum). Daily health checks were performed (at 10 a.m. every day).

### 2.2. Experimental Design

**Figure 1:**
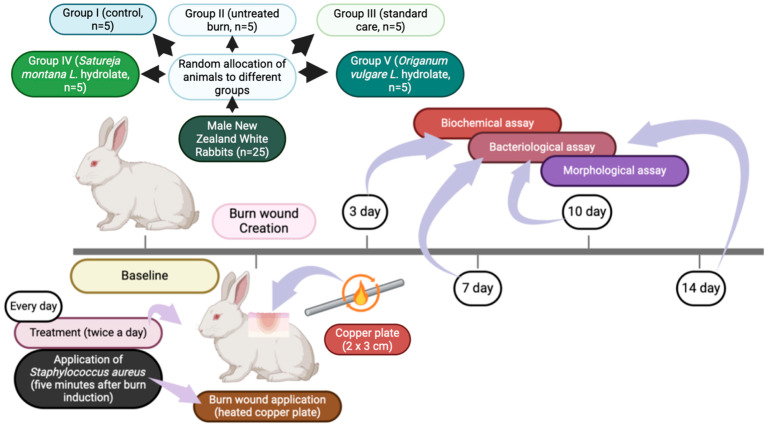
Experimental study design.

The experimental design is schematically illustrated in Figure 1. A total of 25 rabbits were randomly allocated into five distinct experimental groups (n = 5 biological replicates per group). The experimental timeline spanned 14 days post-injury, with key assessments performed on days 3, 7, 10 and 14 to capture the dynamic phases of wound healing.

Experimental groups include:

- Group I (Sham control): Intact, uninjured animals serving as a baseline physiological reference;
- Group II (Burn-Infection control): Animals subjected to burn injury and subsequent bacterial inoculation, receiving no therapeutic intervention;
- Group III (Standard-of-care intervention): Animals with infected burn wounds treated with a standard therapeutic regimen/or «comparative therapy» (boric acid and gauze dressings soaked in Betadine and Levomecol);
- Group IV (*Satureja montana* hydrolate intervention): Animals with infected burn wounds treated with the *Satureja montana* hydrolate-based gel;
- Group V (*Origanum vulgare* hydrolate intervention): Animals with infected burn wounds treated with the *Origanum vulgare* hydrolate-based gel.

Following a 7-day acclimatization period, rabbits were sedated via neuroleptanalgesia (Zoletil^®^ 100, Virbac, Carros, France; 5 mg/100 g body weight, intramuscularly). The dorsal surface was shaved and disinfected. Standardized full-thickness (grade IIIa) thermal burns were induced using a pre-heated copper plate (2 × 3 cm, 200 g), brought to a red-hot state over an alcohol flame. The plate was applied with a controlled pressure of 2 Newtons for 30 s to the pre-shaved dorsal skin. The burn technique was tested and described in our previous pilot work [27]. To facilitate longitudinal monitoring within each animal, four identical burn wounds were created sequentially along the dorsum, extending from the interscapular region caudally.

Immediately following burn induction (within 10 min), each wound surface was inoculated with 0.1 mL of a *Staphylococcus aureus* culture (1.0 McFarland standard, corresponding to 10^8^–10^9^ CFU/mL), prepared in meat-peptone broth. Post-inoculation, wounds were covered with sterile dressings to prevent environmental contamination and ensure consistent bacterial exposure. The severity of grade IIIa burns was confirmed visually by the immediate formation of a distinct, dry, leathery eschar, loss of skin elasticity, absence of immediate pain response upon light touch, initial white/charred tissue appearance, and delayed blister formation, consistent with established literature criteria [28].

Primary criteria included:

1. Eschar formation: A distinct, dry, leathery eschar was formed within hours of burn injury, indicative of coagulation necrosis characteristic of third-degree burns. The eschar was uniformly dense, indicating the depth of the burn extending into the dermis;
2. Loss of skin elasticity and pliability: The burned area had dramatically lost elasticity and was stiff to the touch. This change is characteristic of deeper burns when the collagen fibers of the dermis undergo denaturation;
3. There is no immediate pain response: There was no pain response on light touch or careful manipulation of the burn area, indicating nerve damage in the dermis, a hallmark of third-degree burns. However, the surrounding tissues showed increased sensitivity, consistent with partial-thickness damage;
4. Color change in the burned tissue: Initially, the wound site appeared white or charred, indicating coagulation damage to the dermis and epidermis, in contrast to the redness characteristic of superficial burns;
5. Delayed blister formation: Unlike superficial burns, in which fluid-filled blisters form almost immediately, stage III burns have a delayed blister formation.

Topical application of the respective hydrolate-based gels or «comparative» therapy commenced on the first day post-burn induction and was performed twice daily (morning and evening, with a 10 h interval between applications).

Throughout the experimental period, animals underwent rigorous daily monitoring for general health, behavioral changes, appetite, water intake, and rectal temperature.

**Figure 2:**
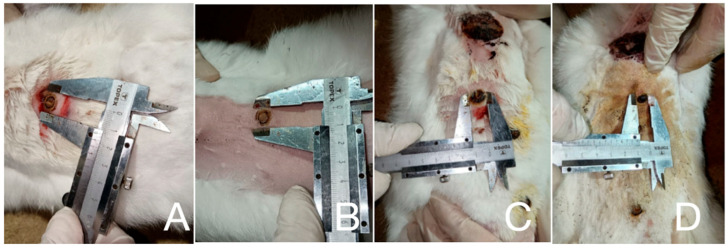
The process of measuring different parameters (length, diameter, width) of the burn injury in different groups (on day 7): (**A**)—Treatment with *Satureja montana* hydro-gel; (**B**)—Treatment with *Origanum vulgare* hydro-gel; (**C**)—Treatment with boric acid with Betadine and Levomecol; (**D**)—Without treatment.

Wound healing progression was meticulously assessed based on criteria including scab formation, reduction in edema, granulation tissue development, onset of marginal epithelialization, and time to complete wound closure (Figure 2). Particular attention was paid to the development of infectious complications.

Animals were humanely euthanized at predetermined time points (days 3, 7, 10 and 14 post-injury) for tissue collection, or earlier if severe septic dermatitis or unresponsive complications necessitated humane removal from the study, in accordance with ethical guidelines.

Euthanasia was performed by intravenous administration of an overdose of sodium pentobarbital (150 mg/kg), in accordance with the AVMA Guidelines for the Euthanasia of Animals (2020) and the institutionally approved animal use protocol. Death was confirmed by the absence of both heartbeat and corneal reflex prior to tissue collection.

Blood samples were collected from the marginal ear vein of each rabbit 24 h prior to burn injury induction (baseline) and on all experimental time points.

Biopsy samples for morphological and immunohistochemical examinations were collected from the injured areas. Prior to biopsy, wound cleaning involved careful debridement of necrotic tissue using surgical tweezers, scalpel, and fine-point scissors to separate the eschar from underlying tissues. Following biopsy, wounds were treated once with a 0.5% chlorhexidine (RX Marine International, Mumbai, India) solution to prevent secondary bacterial contamination (strictly the edges of the wound, without direct treatment of the inner surface of the wound).

### 2.3. Plants Material and Hydrolate Extraction

The botanical identity of *Satureja montana* and *Origanum vulgare* plant species was confirmed through taxonomic authentication by a certified botanist, with corresponding voucher specimens at the herbarium of the Institute of Translational Medicine and Biotechnology, Sechenov University, Moscow, Russia. Hydrolates were subsequently produced via a controlled steam distillation process [29].

The plant material used for hydrolate production was collected during the natural flowering period of both species (June–August) on the southern coast of the Crimean Peninsula, in the coastal region adjacent to the Black Sea.

Specifically, fresh aerial plant material (3.5 kg) from each species was subjected to distillation with deionized water, maintaining a 1:5 (*w*/*v*) plant-to-water ratio. This procedure was conducted for 3 h at atmospheric pressure (100 °C) using a laboratory-scale Clevenger-type apparatus (Duran Group GmbH, Mainz, Germany) with precise temperature regulation via a heating mantle (IKA-Werke GmbH & Co. KG, Staufen, Germany). The resulting vaporized compounds were efficiently condensed using a Liebig condenser (Schott AG, Mainz, Germany), yielding a biphasic distillate. The aqueous phase, representing the hydrolate, was then carefully separated from the essential oil component utilizing a separatory funnel (Pyrex, Corning Inc., New York, NY, USA) and prepared for subsequent analytical and formulation steps.

Key distillation parameters: duration, temperature, pressure, and the plant-to-water ratio, were meticulously monitored and controlled for reproducibility using a calibrated digital thermometer (Testo SE & Co. KGaA, Lenzkirch, Germany) and a pressure gauge (WIKA Alexander Wiegand SE & Co. KG, Klingenberg, Germany). Each prepared hydrolate batch underwent comprehensive evaluation for physical and chemical attributes: visual assessment for clarity, pH determination using a digital pH meter (Hanna Instruments, Woonsocket, RI, USA) within a specified range of 3.5–6.0, density measurement with a digital densitometer (Anton Paar GmbH, Graz, Austria), and refractive index assessment utilizing an Abbe refractometer (ATAGO Co., Ltd., Tokyo, Japan).

Schematic picture of the distillation process and the quality parameters of the resulting compositions are presented in Supplemental Materials (Support Materials S1 and S2).

### 2.4. Microbiological Purity

Microbiological purity was stringently assessed in accordance with European pharmacopeia standards: involved the application of sterile sampling techniques and subsequent incubation in a microbiological incubator (Thermo Fisher Scientific, Waltham, MA, USA) to determine total bacterial count, as well as the presence of molds and yeasts. Chemical profiling was conducted using gas chromatography–mass spectrometry (GC-MS) on an Agilent 7890A GC system interfaced with a 5975C MSD (Agilent Technologies, Santa Clara, CA, USA). This analysis served to identify and precisely quantify the major volatile constituents, thereby confirming the expected chemical composition and validating the efficiency of the extraction process [30].

### 2.5. Hydrogel Preparation

For the preparation of the therapeutic gels, 2% hydroxyethyl cellulose (HEC; Sigma-Aldrich, St. Louis, MO, USA) was precisely weighed to achieve a final concentration of 20 g/L (2% *w*/*v*). This polymer was gradually dispersed into the validated hydrolates (*Satureja montana* and *Origanum vulgare*), which served as the sole solvent (100% aqueous phase) for the hydrogel. This formulation ensures that the active components of the hydrolates are fully integrated into the gel matrix.

This polymer was gradually dispersed into the validated hydrolates under continuous magnetic stirring (IKA-Werke GmbH & Co. KG, Staufen, Germany) at ambient room temperature. To facilitate complete hydration and achieve optimal homogeneity, the mixture was gently heated to 40 °C using a water bath (Julabo GmbH, Seelbach, Germany), with temperature meticulously monitored by a digital thermometer (Testo SE & Co. KGaA, Lenzkirch, Germany). The resulting gels, characterized by their transparent to slightly opalescent appearance, were aseptically transferred into sterile, airtight containers. This transfer was performed under laminar flow conditions within a sterile hood (Thermo Fisher Scientific, Waltham, MA, USA) to prevent any microbial contamination. The final gel products were then stored at 4 °C in a pharmaceutical refrigerator (Liebherr Group, Bulle, Switzerland) to maintain their stability, microbial purity, and bioactivity throughout the entire experimental duration. The final hydrogel formulations exhibited a pH range of 5.5–6.0 and a viscosity of 15,000–20,000 cP (measured at 25 °C), ensuring optimal biocompatibility and application properties.

### 2.6. Bacteriological Assay

All procedures were performed under aseptic conditions within a class II laminar flow cabinet (Model MSC Advantage 1.2, Thermo Fisher Scientific, Waltham, MA, USA) to ensure sterility and prevent contamination.

The in vitro antimicrobial activity of the hydrosol-based gels was evaluated by the microdilution method to determine the minimum inhibitory concentration (MIC) against *Staphylococcus aureus* (ATCC 25923). Serial twofold dilutions of the test samples (pure hydrolates, hydrogel base (2% HEC in water), and final hydrogels) were prepared in Mueller–Hinton Broth (MHB). The concentration range for the hydrolates and hydrogels was from 50% (*v*/*v*) down to 0.09% (*v*/*v*). Bacterial suspensions were adjusted to 10^6^ CFU/mL. After incubation at 37 °C for 24 h, optical density was measured spectrophotometrically at 600 nm to identify the lowest concentration inhibiting visible growth (MIC). The assay was performed in three independent biological replicates, each with three technical replicates. Vancomycin was used as a positive control, and MHB with and without bacteria served as negative and growth controls, respectively. MBC was determined by plating aliquots from all wells showing no visible growth onto Mueller–Hinton Agar and incubating for an additional 24 h.

For the in vivo assessment, tissue biopsy specimens obtained from treated wounds were aseptically homogenized in sterile saline, serially diluted, and plated onto nutrient agar. Triplicates were used for each dilution to ensure reliability and accuracy. Plates were incubated at 37 °C for 24–48 h, after which bacterial colonies were counted. Results were expressed as colony-forming units per gram of tissue (CFU/g).

### 2.7. Biochemical Assay

For analysis, serum was collected in plain tubes after clotting and centrifugation at 3000 rpm for 12 min. All aliquots were stored at −80 °C until use.

Serum samples were analyzed for a panel of standard biochemical markers, including hepatic (alanine aminotransferase (ALT), aspartate aminotransferase (AST)) and renal (creatinine, blood urea nitrogen (BUN)) function indicators, using a Cobas^®^ 8000 modular analyzer system (Roche Diagnostics, Basel, Switzerland). All analyses were performed according to manufacturer’s specifications, with quality control materials analyzed at each session.

### 2.8. Morphological Assay

For a morphological assessment of the condition of the burn wound, fragments of the affected skin were fixed in a solution of buffered formalin, and then wiring (tissue histological wiring apparatus, Leica Biosystems, Hamburg, Germany) was embedded into paraffin blocks, from which serial sections (3 microns thick) were prepared, dewaxed, dehydrated, and stained with Mayer hematoxylin and eosin [31].

The morphometric assessment included quantitative evaluation of total skin thickness, epidermal thickness, stratum corneum thickness, dermal layer thickness, and the dermal repair index (DRI) on H & E-stained sections. Measurements were performed in ImageJ (v1.54f). For each image, the spatial scale was calibrated using the built-in stage micrometer tool. From the central wound zone (avoiding contraction edges and adnexal islands), five non-overlapping regions of interest were analyzed per section and averaged to obtain one value per animal. Data were tabulated in Microsoft Excel (Office 2024).

Linear thickness measurements:

1. Total skin thickness (µm): from the outer epidermal surface to the lower border of the reticular dermis (upper margin of subcutis/panniculus carnosus in rabbits);
2. Epidermal thickness (µm): from the basal membrane to the top of the stratum corneum;
3. Stratum corneum thickness (µm): measured separately as the keratinized layer;
4. Dermal layer thickness (µm): papillary + reticular dermis.

Dermal Repair Index (DRI): DRI quantifies the proportion of new fibrovascular granulation tissue within the dermis and serves as an inverse proxy of dermal maturation/edema. In each region of interest, granulation tissue was identified as the cell-rich, capillary-dense fibrovascular layer beneath the wound surface (including edema within this layer, excluding superficial fibrin/pus crusts). Total dermal thickness was measured in the same region of interest (papillary to reticular dermis). DRI was calculated as:

(1) $$DRI\;\left(\%\right)=\frac{t_{granulation}}{t_{dermis,\;total}}\times 100;$$

where t_granulation_ is the thickness of the fibrovascular granulation layer (µm) and t_dermis,total_ is total dermal thickness (µm). Lower DRI indicates more advanced remodeling (compact collagen, less edema); higher DRI indicates immature, edematous granulation.

All measurements were performed independently by two blinded observers. Inter-observer variability was maintained <5% for all parameters.

### 2.9. Immunohistochemical Assay

An immunohistochemical study was performed according to the standard protocol: sections were heated manually in a steamer with R-UNIVERSAL epitope recovery buffer (Aptum Biologics Ltd., Southampton, UK, SO16 8AD) at 95 °C × 30 min and quenched with endogenous peroxidase. Primary antibodies were applied first, followed by secondary antibodies. Monoclonal antibodies to the proliferation factor Ki-67 (ThermoFisher, Agawam, MA, USA; Clone SP6), the pan-macrophage (M1) marker CD68 (ThermoFisher, Agawam, MA, USA; Clone KP1), and the M2-macrophage marker CD163 (ThermoFisher, Agawam, MA, USA; Clone 10D6) were used as primary antibodies for multiplex immunofluorescence. For secondary antibody detection, a Tyramide Signal Amplification (TSA)-based Opal 7-color Automation IHC kit (Akoya Biosciences, Marlborough, MA, USA) was employed, with each primary antibody paired with a distinct Opal fluorophore (Opal 520, Opal 570, Opal 690). All staining was performed on a fully automated Bond RX autostainer (Leica Biosystems, Lincolnshire, IL, USA) to ensure high throughput and reproducibility. The final concentration of primary and secondary antibodies was meticulously titrated to determine optimal signal-to-noise ratios, typically ranging from 1:50 to 1:200 for primary antibodies. Cell nuclei were stained with Mayer’s hematoxylin. In multiplex immunohistochemistry, nuclei were detected using DAPI (Sigma, Steinheim, Germany; D9542-5MG) for nuclear counterstaining in multiplex immunofluorescence.

Histological and immunohistochemical analyses were performed using a video microscopy system (Leica DM3000 microscope, Hamburg, Germany; DFC450 C camera (Leica Microsystems, Wetzlar, Germany), Platrun LG computer (LG, Seoul, Republic of Korea) and Leica Application Suite (LAS) Version 4.9.0 image processing and analysis software. The number of immunopositive cells was counted in 10 randomly selected fields of view at ×400 magnification (in %). Post-multiplex slides were viewed on a ZEISS Axio Imager.Z2 equipped with a Zeiss alpha Plan-Apochromat (Carl Zeiss AG, Oberkochen, Germany) 100×/1.46 Oil DIC M27 objective lens, a Zeiss Objective Plan-Apochromat 150×/1.35 Glyc DIC Corr M27 objective lens, and a ZEISS Axiocam 712 color digital microscope camera. Images were processed using the Zen 3.0 Light Microscopy Software Package”, “ZEN Module Bundle Intellesis & Analysis for Cells 2022, 11, ×Light Microscopy”, and “ZEN Module Z Stack Hardware” (Carl Zeiss Vision, Jena, Germany).

The proportion of Ki-67-positive cells in the epidermis per unit area, and the absolute number of CD68+ and CD163+ macrophages in the dermis, were determined using the open-source digital image analysis software QuPath (Ver. 0.5.0) to precisely quantify cellular phenotypes and proliferation indices.

### 2.10. Statistical Analysis

Statistical analysis of the sample was performed using STATISTICA 13.5.0.17 software (TIBCO Software Inc., Palo Alto, CA, USA). The Shapiro–Wilk test was used to assess the normality of the data distribution.

For comparisons between study groups with non-normal distributions, the Kruskal–Wallis test followed by Dunn’s post hoc test was applied. Effect sizes (epsilon-squared for Kruskal–Wallis and rank-biserial correlation for Mann–Whitney U test) and, where appropriate, bootstrapped confidence intervals or interquartile ranges, were calculated to provide a comprehensive understanding of the magnitude and precision of observed effects. Parameters exhibiting a normal distribution were analyzed using a two-way Analysis of Variance (ANOVA) followed by Tukey’s post hoc test for multiple comparisons. Parameters with a non-normal distribution were analyzed using the non-parametric Kruskal–Wallis test followed by Dunn’s post hoc test. Multiple comparisons were performed using the Mann–Whitney U test. A *p*-value ≤ 0.05 was considered statistically significant.

## 3. Results

### 3.1. Bacteriological Analysis

The in vitro antimicrobial activity of the *Lamiaceae* hydrolates and their corresponding hydrogel formulations was evaluated against *Staphylococcus aureus* (ATCC 25923) using the microdilution method to determine the minimum inhibitory concentration (MIC) and minimum bactericidal concentration (MBC).

The hydrogel base (2% HEC in water) exhibited no antimicrobial activity against *S. aureus* at the highest tested concentration (50% *v*/*v*), confirming that the observed effects are solely attributable to the incorporated hydrolates. The positive control, Vancomycin, demonstrated high efficacy with an MIC of 1.0 µg/mL and an MBC of 2.0 µg/mL, validating the sensitivity of the bacterial strain and the reliability of the assay.

Both *Satureja montana* and *Origanum vulgare* hydrolates demonstrated antimicrobial activity. The pure *S. montana* hydrolate (SMH) showed an MIC of 6.25% (*v*/*v*) and an MBC of 12.5% (*v*/*v*). The *O. vulgare* hydrolate (OVH) was slightly less potent, with an MIC of 12.5% (*v*/*v*) and an MBC of 25.0% (*v*/*v*). The MBC/MIC ratio of 2.0 for both pure hydrolates indicates a primarily bactericidal mode of action.

The final hydrogel formulations, which use the pure hydrolates as their aqueous phase, maintained the potent antimicrobial properties. The SMH-Hydrogel demonstrated an MIC of 6.25% (*v*/*v*) and an MBC of 12.5% (*v*/*v*). Similarly, the OVH-Hydrogel showed an MIC of 12.5% (*v*/*v*) and an MBC of 25.0% (*v*/*v*).

For the in vivo evaluation, bacterial load in wound tissue biopsies was quantified at various time points (Table 1). At baseline (post-infection, pre-treatment), all infected groups (Groups II–V) exhibited high bacterial counts, with no significant differences among them (approximately 10^7^ CFU/g tissue). This confirmed establishment of the *Staphylococcus aureus* infection model across these groups.

By day 3 post-injury, burn-infection control group (Group II) showed a persistent high bacterial load, averaging 9.8 × 10^6^ CFU/g. The standard care group (Group III) demonstrated a modest reduction to 5.1 × 10^6^ CFU/g, indicating some efficacy of conventional treatment. Notably, both hydrolate-treated groups exhibited reductions in bacterial counts: group IV (*Satureja montana* hydrolate intervention) showed a bacterial load of 2.3 × 10^6^ CFU/g, while Group V (*Origanum vulgare* hydrolate intervention) recorded 3.9 × 10^6^ CFU/g. These reductions were statistically significant compared to Group II (*p* = 0.041 and *p* = 0.035, respectively).

By day 14, the *Satureja montana* hydrolate intervention group exhibited near-complete eradication of *S. aureus* from the wound tissue, with bacterial counts dropping to 1.21 × 10^4^ CFU/g, approaching the levels observed in uninfected control tissues (Sham control). This represented a statistically reduction compared to all other infected groups (*p* = 0.0008). *Origanum vulgare* hydrolate intervention group also showed long-term control, with bacterial loads of 8.0 × 10^4^ CFU/g, lower than burn-infection control group and standard-of-care intervention group (*p* = 0.009). Group III maintained a moderate bacterial load of 1.8 × 10^6^ CFU/g, while Group II continued to show high bacterial persistence (7.0 × 10^6^ CFU/g).

The sham control group consistently showed no detectable bacterial growth throughout the study.

### 3.2. Biochemical Assay

At baseline all experimental groups (I–V) demonstrated comparable levels of serum biochemical parameters (ALT, AST, creatinine, BUN); this confirmed the absence of any pre-existing hepatic or renal impairment. Animals (n = 2) that showed clinically heterogeneous biochemical and actual responses were replaced with rabbits demonstrating normal responses.

The control group (Sham control) maintained stable values throughout the experiment and was used as a physiological reference. Biochemical markers were evaluated at all time points (however, we want to show the three main stages: acute, subacute and recovery phases of the burn-induced systemic response, so we do not treat the 10-day period separately in this section).

On day 3 post-injury, the burn-infected control (group II) demonstrated a clear hepatic and renal response reflected by an increase in ALT (72 ± 4.4 U/L) and AST (86 ± 3.6 U/L) activity as well as by elevated creatinine (1.4 ± 0.1 mg/dL) and BUN (20.1 ± 1.5 mg/dL). But the observed rise, values remained within subclinical limits, suggesting functional rather than structural disturbance. In contrast, animals receiving hydrolate-based treatment exhibited markedly lower deviations of hepatic and renal markers at the same time point (vs. animals subjected to burn injury and subsequent bacterial inoculation, receiving no therapeutic intervention), which may indicate partial attenuation of systemic inflammatory burden. The standard care group showed intermediate results with moderate improvement compared to untreated animals.

By day 7, the most pronounced normalization was observed in animals treated with *Satureja montana* hydrogel (group IV), where ALT and AST activity decreased to 40 ± 1.9 and 52 ± 0.8 U/L, respectively, and renal indices (creatinine 1.2 ± 0.2 mg/dL; BUN 18.3 ± 0.5 mg/dL) closely approximated control levels. The *Origanum vulgare* (group V) showed similar trends with slightly less pronounced improvement, whereas the standard-of-care intervention group demonstrated delayed reduction. The burn-infected control group still exhibited moderate elevation of hepatic enzymes and nitrogenous metabolites (indicating an ongoing but diminishing systemic response to injury and infection).

By day 14, serum biochemical parameters in all treated groups were within normal limits and did not differ significantly from the control. *Satureja montana* group maintained the most stable profile, with ALT and AST values returning to baseline (38 ± 2.1 and 50 ± 1.2 U/L, respectively) and renal indices (creatinine 1.1 ± 0.1 mg/dL; BUN 17.4 ± 0.6 mg/dL) fully normalized. The data is collected in the Table 2.

### 3.3. Macroscopic Examination

Macroscopic evaluation of burn wounds in rabbits infected with *S. aureus* revealed expected differences in healing progress in the experimental groups and over all time points.

In the burn-infection control group, the burn surface remained covered with a thick leathery scab surrounded by pronounced erythema and edema during the first week. By the third day, the wound bed was dull brown in color and dry (all animals had viscous yellowish/brownish exudate with a pronounced unpleasant odor). In contrast, in the standard-of-care intervention group, there was a partial reduction in edema and exudation, although the scab remained.

In the groups receiving hydrogels based on hydrolate, the wound surface appeared noticeably cleaner and more moisturized: in the *Satureja montana* group, the scab was thinner and by day 7 began to peel off spontaneously, exposing bright red granulation tissue with early contraction, while the *Origanum vulgare* group showed a similar, albeit slightly delayed, improvement by day 10.

By day 10, *Satureja montana* hydrolate intervention (group IV) showed almost complete macroscopic closure with smooth pale pink neoepithelium and no visible exudate, while *Origanum vulgare* hydrolate intervention (group V) showed extensive epithelial union with minimal crust remnants. By day 14, complete wound closure and normal skin elasticity were achieved in group IV, accompanied by the absence of secondary infection; partial closure was observed in group V and the standard treatment group, while untreated animals continued to have purulent crusts and delayed contraction.

### 3.4. Morphological Assay

**Figure 3:**
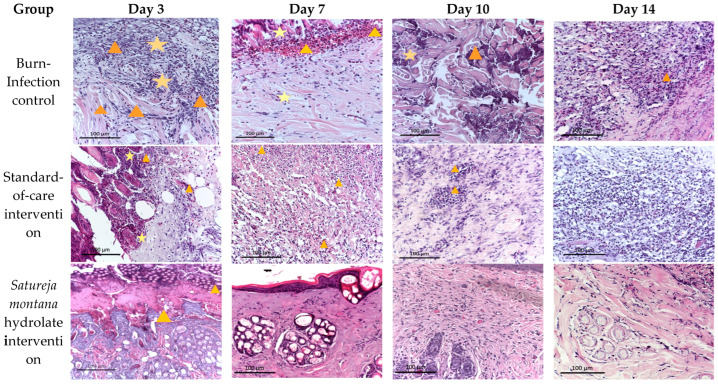

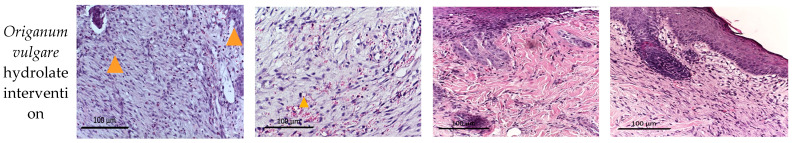
Morphological picture of burn wounds on the skin of rabbits in all experimental groups on different days of the experiment. Stained with H & E, magn. ×200. Symbols (captions to figures): ⋆—detritus, ∆—inflammation.

Histological assay confirmed marked differences in tissue response among the groups over all time points (Figure 3). For example, on the third day, untreated infected wounds showed extensive coagulative necrosis of the epidermis and upper dermis with dense neutrophilic infiltration, vascular congestion, and clusters of *S. aureus* within necrotic debris. The dermal matrix was highly disorganized, reflecting an uncontrolled inflammatory phase.

In contrast, animals receiving standard therapy or *Origanum vulgare* hydrogel exhibited partial demarcation of necrotic tissue and early fibroblast migration, while the *Satureja montana* group already displayed cleaner wound margins and emerging granulation tissue with fewer neutrophils.

By day 7, the differences became more pronounced. Untreated wounds remained purulent and edematous with persistent bacterial contamination, whereas standard-treated and *O. vulgare* groups showed moderate granulation and incomplete epithelial migration. In the *S. montana* group, well-formed granulation tissue rich in fibroblasts and new capillaries were evident, and the inflammatory infiltrate had shifted toward mononuclear cells, indicating suppression of infection and transition to active repair.

On day 10, untreated burns still contained necrotic fragments and mixed inflammatory foci. The standard-care and *O. vulgare* groups demonstrated partial epithelial coverage over immature stroma. In contrast, *S. montana*-treated wounds showed an almost continuous multilayered epithelium, compact collagen deposition, and minimal inflammation.

By day 14, the *S. montana* hydrogel group exhibited complete structural recovery: mature keratinized epithelium, organized collagen bundles, and restored adnexal elements; while *O. vulgare* and standard therapy achieved partial regeneration with residual lymphoid infiltration. The untreated infected group remained incompletely epithelialized, with fibrosis and chronic suppurative inflammation.

**Figure 4:**
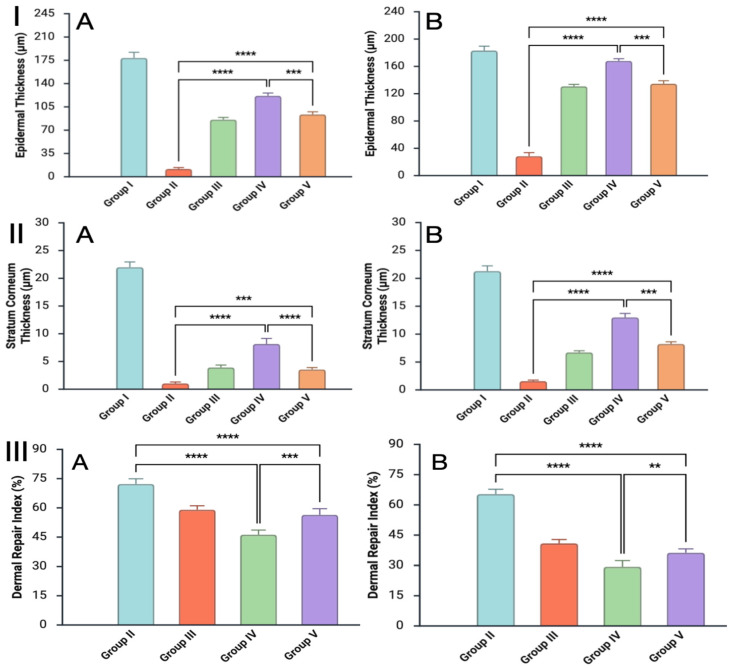
Dynamics of epidermal and dermal recovery in *S. aureus*–infected rabbit skin following treatment with hydrolate-based hydrogels. (**I**)—epidermal thickness; (**II**)—stratum corneum thickness; (**III**)—dermal repair index (DRI). Panels (**A**,**B**) correspond to days 7 and 14 post-burn. Bars represent mean ± SE; statistical significance indicated as ** *p* < 0.01; *** *p* < 0.001, **** *p* < 0.0001. The names of the groups correspond to the study design: group I—sham control; group II—burn-Infection control; group III—standard-of-care intervention; group IV—*Satureja montana* hydrolate intervention; group V—*Origanum vulgare* hydrolate intervention.

Quantitative morphometric assessment was performed on histological sections obtained on the 7th and 14th days after burn induction to evaluate the progression of epidermal and dermal repair (Figure 4).

Three key parameters were analyzed: (1) Epidermal thickness (μm)—from the basement membrane to the stratum corneum; (2) Stratum corneum thickness (μm) and (3) Dermal repair index (DRI), calculated as the ratio of the thickness of newly formed granulation tissue to the total thickness of the dermis (expressed as a percentage), reflecting the degree of dermal remodeling.

On day 7, the non-treated infected wounds showed almost complete epidermal loss (mean thickness < 30 µm) and high DRI values (>60%), indicating massive edema and unstructured granulation. The standard-care therapy and *Origanum vulgare* groups displayed partial epidermal recovery (85–95 µm) with early keratinization, while *Satureja montana*-treated wounds demonstrated substantially greater epidermal thickness (approximately, 120 µm) and a lower DRI (45%), signifying more compact, organized granulation tissue.

By day 14, the difference between groups became more evident. Infected untreated wounds remained atrophic (app., 35 µm epidermis) with a disrupted stratum corneum and residual inflammation. The standard-care and *O. vulgare* hydrogels promoted significant epidermal recovery (130–145 µm) and partial normalization of the stratum corneum (6–9 µm). The *S. montana* group reached near-physiological epidermal thickness (appr., 175 µm) and stratum corneum values comparable to healthy skin (19 µm). Its DRI dropped to 28%, consistent with mature dermal remodeling and minimal edema.

### 3.5. Immunohistochemical Assay

**Figure 5:**
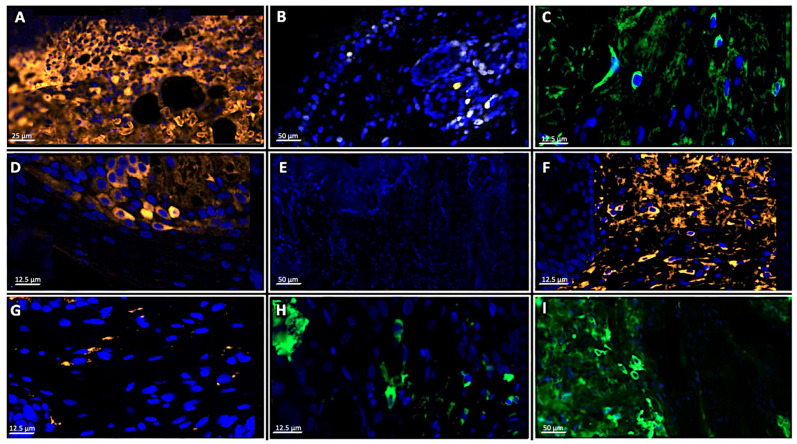
Multiplex analysis of cellular proliferation and macrophage polarization in burn wounds: (**A**)—Sham group skin on day 3; (**B**)—*Satureja montana* group (Group IV) on day 3; (**C**)—Standard-of-care intervention group on (Group III) on day 14 (showing CD163+ M2 macrophages (green) in the papillary layer of the dermis); (**D**)—Standard-of-care intervention group on (Group III) on day 3; (**E**)—Burn-Infection control group (Group II) on day 3 (minimal Ki-67+ proliferative activity); (**F**)—Burn-Infection control group (Group II) on day 7 (high density of CD68+ macrophages (orange); (**G**)—*Satureja montana* group (Group IV) on day 14; (**H**)—Burn-Infection control group (Group II) on day 7 (CD163+ M2 macrophages (green) within the inflammatory infiltrate); (**I**)—*Origanum vulgare* group (Group V) on day 14 (CD163+ M2 macrophages (green) in the reparative tissue). Representative immunofluorescence images of skin sections from experimental groups at various time points, stained for cellular proliferation (Ki-67, yellow/white signal), pan-macrophage infiltration (CD68, orange signal), and M2-macrophage polarization (CD163, green signal). Cell nuclei are counterstained with DAPI (blue signal), magn. ×40 for all panels.

**Figure 6:**
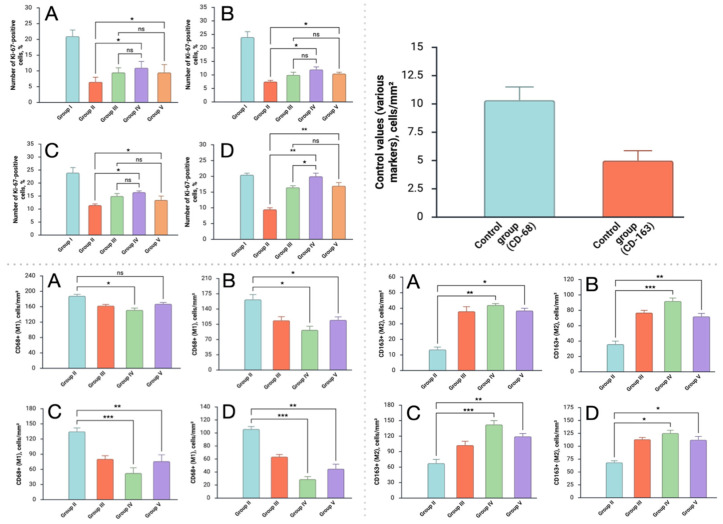
The number of Ki-67-, CD68-, CD163-positive-cells in the epidermis of the control and experimental groups, (in % and cells/mm^2^) at various study stages: (**A**) 3rd day, (**B**) 7th day, (**C**) 10th day, (**D**) 14th day. The experimental groups are numbered according to the design of the study. Bars represent mean ± SE; statistical significance indicated as ns—not significant; * *p* < 0.05; ** *p* < 0.01; *** *p* < 0.001. The names of the groups correspond to the study design: group I—sham control; group II—burn-Infection control; group III—standard-of-care intervention; group IV—*Satureja montana* hydrolate intervention; group V—*Origanum vulgare* hydrolate intervention.

The dynamic cellular response to burn injury and subsequent therapeutic intervention was quantitatively and qualitatively assessed using multiplex immunofluorescence targeting the proliferation marker Ki-67, the pan-macrophage/M1 marker CD68, and the M2-macrophage marker CD163 (Figure 5 and Figure 6). Macrophage polarization was analyzed by tracking the shift from the pro-inflammatory M1 phenotype (CD68-dominant) to the pro-resolving/reparative M2 phenotype (CD163-dominant).

On Day 3 post-injury, the burn-Infection control group exhibited minimal Ki-67-positive cells in the basal epidermal layer at the wound margins, reflecting the severe, infection-driven and burn suppression of the initial proliferative phase. In contrast, the *Satureja montana* group demonstrated a higher proliferative index (Ki-67 expression) at the epidermal edges, indicating an early initiation of re-epithelialization. The standard-of-care (Group III) and *Origanum vulgare* (Group V) groups showed intermediate Ki-67 activity, slightly higher than Group II but notably less pronounced than Group IV.

Pro-inflammatory dominance all infected groups (II, III, IV, V) showed a high density of CD68+ (M1) macrophages within the inflammatory infiltrate, particularly in the deep dermis and hypodermis. This is consistent with the initial phase of infection and debridement. However, the *Satureja montana* group showed the earliest evidence of M2 polarization, with CD163+ cells beginning to emerge in the papillary dermis. The Burn-Infection control group maintained a nearly exclusive CD68+ population, indicating a sustained and unresolved pro-inflammatory state (Figure 5F, day 7, showing sustained CD68+).

By Day 7, the differences in proliferative activity became more competitive. In Group IV, Ki-67 expression was widespread and highly organized within the migrating epithelial tongue, indicating rapid closure of the wound surface. The *O. vulgare* and Standard-of-care groups showed only localized and less continuous Ki-67 staining, consistent with the partial epithelial coverage observed morphologically. By Day 14, Group IV exhibited a mature, multi-layered epidermis with Ki-67 staining restricted almost exclusively to the basal layer, mirroring the homeostatic proliferative pattern of intact skin. Conversely, the other groups, particularly Group II, still showed disorganized or minimal Ki-67 expression, reflecting incomplete or arrested repair.

In the *Satureja montana* group, there was a pronounced shift, with CD163+ (M2) macrophages becoming the dominant population within the granulation tissue. These M2 cells were spatially associated with areas of active collagen deposition and neovascularization. In contrast, the Burn-Infection control group still displayed a high density of CD68+ macrophages, signifying a pathological persistence of the M1 phenotype, which actively inhibits fibroblast function and promotes tissue destruction. The *O. vulgare* and standard-of-care groups showed a slower, less complete transition, with a mixed M1/M2 population, suggesting a delayed resolution of inflammation.

By the final time point, the *Satureja montana* group showed a reduction in the overall number of macrophages, with the few remaining cells predominantly expressing CD163 (M2), localized to the remodeling dermal matrix. The *Origanum* vulgare group also showed M2 dominance, but with a higher residual density of both M1 and M2 cells, correlating with the residual lymphoid infiltration and less mature tissue structure observed morphologically. The burn-infection control group continued to show a persistent, albeit reduced, inflammatory infiltrate with a higher-than-expected M1 component, consistent with chronic, non-healing wounds.

## 4. Discussion

In the present work, we investigated the complex pathology of burn wounds exacerbated by *Staphylococcus aureus* infection, alongside exploring the therapeutic potential of novel hydrolate-based gels derived from *Satureja montana* and *Origanum vulgare*. Our primary objective was to elucidate the impact of these phytopreparations on critical aspects of wound healing and tissue regeneration within this challenging clinical context.

The integumentary system, primarily constituted by the skin, stands as the human body’s largest organ, indispensable for maintaining homeostasis through its roles in environmental defense, thermoregulation, and sensory transduction [32]. Given its extensive exposure, the skin is frequently vulnerable to various forms of trauma, among which thermal injury, commonly referred to as burns, represents a particularly severe and multifaceted assault. The following insult initiates a profound and dynamic cascade of morphological, cellular, and molecular alterations that extend far beyond the immediate physical destruction, critically influencing both local tissue viability and systemic physiological responses [33]. A critical aspect of this pathology is the development of distinct, concentric zones of tissue damage, a concept foundational to burn pathology (Jackson’s zones), which is further detailed in the Supplementary Materials (Support Material S3).

In the context of our findings, this tripartite concept provides an essential morphological framework for interpreting the divergent outcomes observed across experimental groups. The persistence of coagulative necrosis and neutrophilic infiltration in untreated infected burns supports the notion that the stasis zone undergoes rapid secondary conversion when vascular perfusion and immune regulation are not restored. Histologically, this was reflected by the dominance of polymorphonuclear leukocytes, endothelial congestion, and disorganized dermal collagen (changes that indicate an uncontrolled inflammatory loop rather than a regenerative one).

Beyond the intrinsic pathophysiological responses to thermal injury, the compromised cutaneous barrier renders burn wounds highly susceptible to microbial colonization and subsequent infection, which critically impedes healing and significantly elevates morbidity and mortality [34]. Among the myriad opportunistic pathogens, *Staphylococcus aureus* (*S. aureus*) consistently emerges as a predominant early colonizer and a major etiological agent in burn wound infections [35]. This Gram-positive bacterium presents formidable challenges due to its inherent capacity for robust biofilm formation and the alarming global rise in antibiotic-resistant strains, particularly methicillin-resistant *S. aureus* (MRSA), which profoundly complicates clinical management and necessitates innovative therapeutic approaches (Support Material S4). The progression to invasive infection, often characterized by bacterial loads exceeding 10^5^ colony-forming units per gram of tissue, represents a critical threshold that demands novel, multi-targeted interventions [36,37]. This urgent need for effective strategies against *S. aureus* in the challenging microenvironment of burn wounds forms the central premise for the current investigation into the dual-action potential of *Lamiaceae* hydrolates.

Current burn care protocols emphasize early excision and grafting, infection control, and maintenance of a moist wound environment; however, persistent inflammation and delayed tissue regeneration remain unresolved challenges, underscoring the need for adjunctive therapies that can modulate the wound microenvironment and support intrinsic healing mechanisms [38,39]. This imperative has driven a growing interest in natural products, especially plant-derived compounds, as a rich source of agents with multifaceted biological activities, including antimicrobial, anti-inflammatory, and pro-regenerative properties [40]. These «second-line» or supportive therapies are increasingly recognized for their potential to complement conventional treatments, particularly in mitigating the complex sequelae of burn wounds.

Among the diverse botanical resources, the *Lamiaceae* family, widely known for its aromatic species, has garnered significant scientific attention due to its rich phytochemical profile and established therapeutic potential [41].

*Origanum vulgare* (or oregano) and *Satureja montana* (or mountain savory) are notable members of this family, recognized for their potent biological activities. Their efficacy is largely attributed to the presence of phenolic compounds, predominantly carvacrol and thymol, which are well-documented for their broad-spectrum antimicrobial properties [20,21]. These compounds exert their effects through various mechanisms, including disruption of bacterial cell membranes, inhibition of enzyme activity, and interference with efflux pumps, offering a multi-targeted approach against pathogens like *S. aureus* [25].

Hydrolates, the aqueous co-products generated during the steam distillation of essential oils, represent a sustainable and often less cytotoxic alternative to their concentrated essential oil counterparts. They retain water-soluble aromatic compounds and exhibit significant antimicrobial properties, making them attractive for topical applications [23]. Hydrogels, as biocompatible and moisture-retaining matrices, provide an ideal environment for wound healing, facilitate the sustained release of therapeutic agents, and offer physical protection to the wound bed.

Our own chemical profiling of the *Satureja montana* and *Origanum vulgare* hydrolates (detailed in Supplementary Materials, Tables S1 and S2) revealed a critical compositional difference that underpins our therapeutic findings. Specifically, the *S. montana* hydrolate exhibited a high concentration of carvacrol (86.7%) and a total phenolic content (carvacrol + thymol) of 89.9%. This concentration is higher than the typical carvacrol content reported for many *S. montana* essential oils and hydrolates, and significantly surpasses the total phenolic content (60.8%) observed in our *O. vulgare* hydrolate [42]. This high concentration of the primary antimicrobial agent, carvacrol, in the *S. montana* aqueous phase provides a chemical rationale for its efficacy in the infected burn model, distinguishing our formulation from those based on less concentrated or less potent chemotypes [43].

Our central hypothesis was that hydrogels enriched with *Lamiaceae*-derived hydrolates exert a dual therapeutic effect: direct antimicrobial activity against burn-associated pathogens, including resistant strains like as *Staphylococcus aureus*, and indirect modulation of the inflammatory response that facilitates re-epithelialization and tissue repair.

A clear inverse correlation is observed between the reduction in bacterial load and the improvement in the morphological picture. In burn-infection control group, the persistence of a high *Staphylococcus aureus* titer (10^6^–10^7^ CFU/g) throughout the experiment was accompanied by persistent purulent inflammation, extensive coagulative necrosis, and the absence of organized granulation tissue on days 7 and 14. In contrast, in Group IV (*S. montana*), where near-complete suppression of bacterial contamination (down to 10^4^ CFU/g) was observed by day 14, histological examination showed the formation of mature multilayered epithelium, minimal inflammatory infiltrate, and well-organized collagen bundles. This finding aligns with the propositions put forth by Church et al., who emphasize that a bacterial load above 10^5^ CFU/g of tissue represents a critical threshold that impedes normal healing and necessitates mandatory antimicrobial therapy [44]. Our study gently suggests that the *S. montana* hydrogel demonstrated the ability to effectively overcome this threshold, which may be due to the synergistic action of its phytocomponents on *S. aureus* biofilms, as indicated in the work of Peng et al. [36].

Another important parameter we studied was cell proliferation. The dynamics of cell proliferation and macrophage polarization are closely linked to the phases of wound healing. In *Satureja montana* group, a higher activity of Ki-67-positive cells was noted at the wound edges as early as day 3, indicating an early initiation of re-epithelialization. This coincides with a rapid shift in macrophage phenotype: the *S. montana* group showed a pronounced transition from the pro-inflammatory M1 (CD68-dominant) to the pro-reparative M2 (CD163-dominant) phenotype by day 7. Rose et al., point out that successful burn wound healing critically depends on the timely transition from the inflammatory to the proliferative phase, a process regulated precisely by macrophage polarization [45]. Our result, in which the early predominance of M2 macrophages in the *S. montana* group correlates with the best indices of epidermal thickness and Dermal Repair Index (DRI) by day 14, allows us to hypothesize that the winter savory hydrolate possesses a pronounced immunomodulatory potential, contributing to more effective inflammation resolution and transition to the remodeling phase.

The mechanistic difference between hydrolate-based hydrogel and the standard-of-care intervention is profound. Standard care primarily functions as a broad-spectrum antimicrobial agent, often exhibiting dose-dependent cytotoxicity that can impede the delicate cellular processes of the proliferative phase [46]. In contrast, *S. montana* hydrogel acts as a biologically active system that actively regulates the inflammatory microenvironment. In addition, we observed more rapid M1-M2 polarization in our study, which is a sign of regenerative healing and can be considered a therapeutic goal in the treatment of complex wounds (or, for example, larger areas of damage) [47]. We attribute these results in part to the high concentration of carvacrol in *S. montana* hydrolate, which not only destroys the bacterial load but also modulates the host’s immune response.

The improvement in the local healing picture in the hydrolate-treated groups was also reflected in systemic parameters. The increase in ALT and AST activity, as well as nitrogenous metabolites (creatinine, BUN) on day 3 in burn-infection control group, reflected the systemic response to trauma and infection, as is often described in the literature for severe burns [33]. The faster return of these parameters to normal in *Satureja montana* group by day 7, compared to the control and standard therapy groups, may be a preliminary indication of reduced systemic inflammatory burden and, possibly, the presence of a hepatoprotective or nephroprotective effect in the phytopreparation, which warrants further investigation.

Pharmacologically active compounds present in plant raw materials play an important role. For example, carvacrol and thymol are structurally similar phenolic compounds, differing only in the position of the hydroxyl (-OH) group on the aromatic ring. This hydroxyl group is the cornerstone of their biological activity, acting as a potent hydrogen donor and free radical scavenger. This antioxidant capacity is crucial in mitigating the systemic oxidative stress that characterizes severe burns [48]. By neutralizing reactive oxygen species (ROS), these compounds can protect distant organs, from secondary oxidative damage. This directly supports our observation of a faster return to baseline for ALT, AST, and BUN levels.

**Figure 7:**
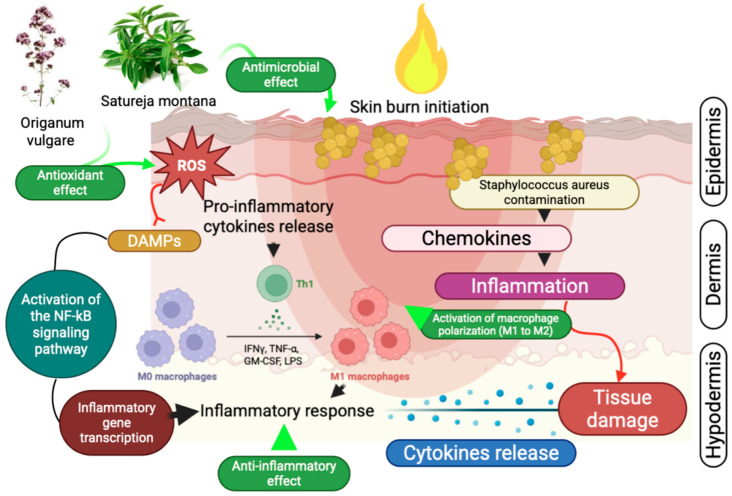
Proposed cellular mechanism underlying the anti-inflammatory and regenerative effects of *Satureja montana* and *Origanum vulgare* hydrolate-based hydrogels in thermal burn injury. The formulations reduce bacterial load and pro-inflammatory cytokines (IL-6, TNF-α, IL-1β), promote M1-to-M2 macrophage polarization (CD68+ → CD163+), enhance keratinocyte proliferation (Ki-67+), and restore antioxidant balance (↑SOD, ↑GSH), leading to accelerated epithelialization and tissue remodeling [11,27,31].

Taken together, the results of our study, demonstrating the pronounced pro-regenerative effect of the *Satureja montana* hydrolate, are generally consistent with the growing body of literature on the use of natural products in burn wound therapy (Figure 7).

The pronounced pro-regenerative effects observed locally (enhanced epithelialization, improved granulation tissue quality) are also deeply rooted in the phytochemical profile of the hydrolates. The primary mechanism is the modulation of the local inflammatory response.

Regarding the *Satureja* genus specifically, our data on its superiority over *Origanum vulgare* in the infected burn wound model in rabbits correlate with the conclusions of Criollo-Mendoza et al., who emphasize that the wound-healing properties of natural products often depend on the precise phytochemical composition, which can vary even between closely related species [49]. In our case, the *S. montana* hydrogel proved to be a highly promising agent, which may be due to the optimal concentration of key bioactive components, known for their antimicrobial and anti-inflammatory properties, in the aqueous phase of the extract.

Carvacrol and thymol are known to inhibit key pro-inflammatory enzymes, like cyclooxygenase-2 (COX-2), and downregulate the production of inflammatory cytokines like TNF-α and IL-1β 6 [48]. This action is critical in the wound microenvironment, as it facilitates a more rapid transition from the pro-inflammatory M1 macrophage phenotype to the pro-reparative M2 phenotype, a switch that we confirmed with our multiplex immunohistochemical analysis (CD68 vs. CD163). This targeted anti-inflammatory action, without the broad immunosuppression of some conventional therapies, creates an ideal environment for tissue regeneration. Our results align with the findings of Farahpour et al., who demonstrated that topical application of another *Lamiaceae* family member, *Salvia officinalis*, accelerated healing in infected wounds by reducing the inflammatory cell infiltrate and promoting collagen deposition [7]. In addition, Anis A. et al., in their study on rabbits, also showed a positive effect of essential oil-containing mixtures on the histopathological parameters of healing, including reduced epithelialization time and improved quality of granulation tissue [40].

The structural difference between carvacrol and thymol, though subtle, can influence their binding affinity to cellular targets and their lipophilicity, potentially explaining the observed difference in efficacy between *S. montana* and *O. vulgare*. For instance, the position of the hydroxyl group in carvacrol may allow for more effective interaction with bacterial membrane proteins or inflammatory pathway enzymes compared to thymol, leading to the superior antimicrobial and anti-inflammatory outcomes seen with the *S. montana* formulation.

Furthermore, when contextualized within the broader literature on herbal hydrogels for burn management, our findings complement and extend existing evidence. Numerous botanicals (most notably *Aloe vera* and *Calendula officinalis*) have been widely explored for their anti-inflammatory and antioxidant potential, and these studies have laid important groundwork for the development of plant-based wound therapies [50]. However, many of these reports primarily describe general wound-conditioning or soothing effects, with fewer studies providing in-depth quantitative insight into specific immunological pathways.

In this regard, our work contributes an additional mechanistic layer to the field. Specifically, we document a measurable shift in the M1/M2 macrophage ratio within a *S. aureus*–infected burn model, suggesting that *Satureja montana* hydrolate may participate in modulating early inflammatory responses. While we do not claim exclusivity in this area, such quantitative immune profiling remains relatively uncommon in studies of herbal hydrogels and may help clarify how individual phytochemicals influence wound-healing trajectories.

Taken together, these findings support the growing view that phytochemical-rich hydrolates may serve as functional components in next-generation wound dressings, particularly when accompanied by rigorous chemical and immunological characterization [51].

An important aspect to consider when interpreting our results is the limitation associated with the use of only male rabbits in the study. This approach was consciously chosen to minimize the potential influence of cyclic hormonal fluctuations, characteristic of females, on the dynamics of the inflammatory process and wound healing. As is known, sex hormones, particularly estrogens, can exert a significant modulating effect on the immune response and cell proliferation in the wound, by Günter et al. [41]. By excluding this factor, we were able to more clearly isolate the direct therapeutic action of the investigated hydrogels.

In addition, our study utilized the New Zealand White rabbit model for the in vivo burn infection assessment. This model was deliberately selected due to its well-established utility in burn research, particularly because the dermal structure and thickness of rabbit skin are considered more comparable to human skin than those of smaller rodent models [52,53]. Furthermore, the larger size of the rabbit facilitates the creation of standardized burn wounds and allows for the comprehensive collection of tissue and systemic samples necessary for multiplex analysis (histology, immunohistochemistry, and biochemical assays). However, we acknowledge that no animal model perfectly replicates the human condition.

Nevertheless, to ensure the full translational significance of the data obtained, future research will be directed at addressing this limitation. Specifically, our plans include: expansion of the model to female rabbits to assess potential gender differences in therapeutic efficacy; testing the hydrogels on other animal models (e.g., rats or pigs) to confirm the universality of the therapeutic effect; and in-depth study of the mechanisms of action using in vitro approaches (keratinocyte, fibroblast, and macrophage cultures) to precisely define the molecular targets.

In conclusion, the present work lays the foundation for understanding the complex action of hydrogels based on *Satureja montana* and *Origanum vulgare* hydrolates in the context of an infected burn wound. The data obtained allow us to state gently but confidently that the *Satureja montana* hydrogel presents itself as a highly promising candidate for the development of adjuvant therapy. Further pressure on genetic, molecular, and proteomic investigations, as well as the consideration of this issue from various angles, will undoubtedly help bring the integration of these natural phytopreparations closer to clinical practice.

## 5. Conclusions

The present study successfully investigated the dual modulatory effects of *Lamiaceae* hydrolate-loaded hydrogels on skin repair in *Staphylococcus aureus*-infected burn wounds, with the *Satureja montana* formulation demonstrating the most significant efficacy.

Our comprehensive analysis confirmed that this hydrogel effectively addressed the key challenges of the model: it achieved a near-complete eradication of *S. aureus*, reducing the bacterial load to 1.21 × 10^4^ CFU/g by Day 14, a substantial reduction compared to the untreated control (6.9 × 10^6^ CFU/g). Furthermore, the treatment actively promoted tissue repair, evidenced by the formation of mature epidermis (approximately 175 µm thick) and a favorable shift in the inflammatory microenvironment, characterized by an early transition to the pro-reparative M2 macrophage phenotype. In summary, the *Satureja montana* hydrogel presents itself as a highly promising candidate for the development of an effective adjunctive therapy, demonstrating a beneficial combination of antimicrobial action and pro-regenerative properties.

Future research should explore optimization of the hydrogel formulation, physicochemical parameters, stability, and bioavailability. In addition, validation of these findings in larger animal models or ex vivo human skin systems would provide an important translational step toward assessing safety, dosing, and real-world applicability.

## Figures and Tables

**Table 1:** Bacterial load (CFU/g tissue) in burn wounds across experimental groups at different time points.

Experimental Group	Baseline	Day 3	Day 7	Day 14
Sham control	ND	ND	ND	ND
Burn-Infection control	1.20 (±0.15) × 10^7^	9.80 (±0.95) × 10^6^	8.52 (±0.88) × 10^6^	6.9 (±0.75) × 10^6^
Standard-of-care intervention	1.09 (±0.12) × 10^7^	5.14 (±0.61) × 10^6^	3.07 (±0.45) × 10^6^	1.88 (±0.32) × 10^6^
*Satureja montana* hydrolate intervention	1.32 (±0.18) × 10^7^	2.32 (±0.30) × 10^6^ (*p* = 0.041)	7.20 (±0.15) × 10^5^ (*p* = 0.009)	1.21 (±0.06) × 10^4^ (*p* = 0.0008)
*Origanum vulgare* hydrolate intervention	1.2 (±0.14) × 10^7^	3.99 (±0.45) × 10^6^ (*p* = 0.035)	1.58 (±0.25) × 10^6^ (*p* = 0.015)	8.01 (±0.12) × 10^4^ (*p* = 0.009)

*Notes.* ND: not detectable. *p*-values are calculated versus Group II (Burn-Infection Control) using the Mann–Whitney U test. All data is presented as Mean ± SD.

**Table 2:** ALT (U/L), AST (U/L), Creatinine (mg/dL), BUN (mg/dL) across all experimental groups at different time points.

Experimental Group	Baseline	Day 3	Day 7	Day 14
Sham control	ALT: 34 ± 2.8 AST: 40 ± 3.6 Creatinine: 0.8 ± 0.2 BUN: 18.4 ± 1.2	ALT: 39 ± 5.1 AST: 37 ± 2.5 Creatinine: 1.1 ± 0.3 BUN: 17.8 ± 0.8	ALT: 36 ± 1.1 AST: 42 ± 1.9 Creatinine: 0.92 ± 0.4 BUN: 18.0 ± 0.8	ALT: 37 ± 2.5 AST: 38 ± 2.0 Creatinine: 0.7 ± 0.1 BUN:18.5 ± 1.0
Burn-Infection control	ALT: 33.6 ± 3.0 AST: 40.4 ± 5.0 Creatinine: 0.82 ± 0.4 BUN: 16.9 ± 2.0	ALT: 72 ± 4.4 * (*p* = 0.033) AST: 86 ± 3.6 ** (*p* = 0.0021) Creatinine: 1.4 ± 0.1 BUN: 20.1 ± 1.5 ** (*p* = 0.0041)	ALT: 52 ± 6.2 * (*p* = 0.031) AST: 61 ± 6.0 ** (*p* = 0.008) Creatinine: 1.6 ± 0.2 BUN: 20.4 ± 1.3	ALT: 37 ± 1.4 AST: 45 ± 4.0 Creatinine: 1.0 ± 0.1 BUN:19.2 ± 1.0
Standard-of-care intervention	ALT: 33.1 ± 4.0 AST: 39.9 ± 3.0 Creatinine: 0.78 ± 0.4 BUN: 17.2 ± 1.5	ALT:58 ± 5.0 * (*p* = 0.027) AST: 73 ± 1.6 Creatinine: 1.2 ± 0.1 ** (*p* = 0.01) BUN:21.5 ± 2.2	ALT: 44 ± 2.1 AST: 56 ± 4.0 Creatinine: 1.4 ± 0.1 BUN: 21.0 ± 1.0	ALT: 38 ± 3.7 AST: 42 ± 3.0 Creatinine: 0.9 ± 0.3 BUN:17.4 ± 0.8
*Satureja montana* hydrolate intervention	ALT: 35.1 ± 1.0 AST: 40.3 ± 4.6 Creatinine: 0.85 ± 0.3 BUN: 18.0 ± 2.0	ALT: 53 ± 7.1 ^#^ (*p* = 0.0049) AST: 66 ± 3.0 ^#^ (*p* = 0.0035) Creatinine: 1.4 ± 0.1 BUN: 20.0 ± 0.5	ALT: 40 ± 1.9 AST: 52 ± 0.8 ^#^ (*p* = 0.003) Creatinine: 1.2 ± 0.2 BUN: 18.3 ± 0.5	ALT: 31 ± 1.2 AST: 46 ± 4.0 Creatinine: 1.1 ± 0.1 BUN:14.4 ± 2.2
*Origanum vulgare* hydrolate intervention	ALT: 38.0 ± 3.0 AST: 32.6 ± 2.6 Creatinine: 1.4 ± 0.2 BUN: 15.3 ± 2.2	ALT: 57 ± 4.5 ^#^ (*p* = 0.0041) AST: 62 ± 2.1 ^#^ (*p* = 0.0021) Creatinine: 1.4 ± 0.3 BUN: 17.4 ± 2.0	ALT: 48 ± 1.0 AST: 48 ± 2.7 ^#^ (*p* = 0.0021) Creatinine: 1.0 ± 0.2 BUN: 19.4 ± 0.7 * (*p* = 0.042)	ALT: 33 ± 2.4 AST: 29 ± 2.0 Creatinine: 0.6 ± 0.2 BUN:16.4 ± 2.5

*Notes.* Statistical analysis was performed using a two-way ANOVA followed by Tukey’s post hoc test for multiple comparisons: * *p* < 0.05, ** *p* < 0.01 vs. Group I (Sham Control). ^#^ *p* < 0.05 vs. Group II (Burn-Infection Control). All data is presented as Mean ± SD.

## Data Availability

The original contributions presented in this study are included in the article/Supplementary Material. Further inquiries can be directed to the corresponding author(s).

## Supplementary Materials

The following supporting information can be downloaded at: https://www.mdpi.com/article/10.3390/antibiotics15010020/s1. Support Material S1. Table S1: Chemical composition of *Satureja montana* hydrolate; Table S2: Chemical composition of *Origanum vulgare* hydrolate; Support Material S2. Figure S1: Distillation unit diagram (hydro-parodistillator): 1—heating element; 2—water drain valve; 3, 4—steam jacket; 5—partition; 6—lid valve; 7—distillation cube lid; 8, 9—steam outlet pipe; 10—steam outlet tube screw; 11—condenser (refrigerator); 12—receiver (Florentine flask); 13—oil container; 14—hydrolates; Support Material S3. Figure S2: Schematic representation of Jackson’s burn wound model, illustrating three concentric zones: the central zone of necrosis, the intermediate zone of stasis, and the peripheral zone of hy-peraemia; Support Material S4. Figure S3: Schematic representation of Staphylococcus aureus infection in burn wounds, illustrating bacterial colonization, biofilm formation, and toxin-mediated immune activation. References [36,37,54,55,56,57,58,59] are cited in the Supplementary Materials.
