# Supplementary material for: Dual Modulation of Infection and Skin Recovery by Lamiaceae Hydrolate Hydrogels in S. aureus-Infected Burns
Source: Antibiotics (Basel). 2025 Dec 22;15(1):20. doi: 10.3390/antibiotics15010020 (PMC12837250; doi:10.3390/antibiotics15010020)
Supplement: Supplementary file 1 [file antibiotics-15-00020-s001.zip › antibiotics-3982239-supplementary.pdf]

DUAL MODULATION OF INFECTION AND SKIN RECOVERY BY LAMIACEAE HYDROLATE-LOADED HYDROGELS IN S. AUREUS-INFECTED BURNS

Grigory Demyashkin, Mikhail Parshenkov, Alibek Tokov, Tatiana Sataieva, Anatoly Kubyshkin, Vladimir Shchekin, Sergey Popov, Boris Kuzminov, Nadezhda Zabroda, Artem Volodkin, Kirill Blinov, Petr Shegay and Andrei Kaprin

Support material S1.

The botanical identity of *Satureja montana* and *Origanum vulgare* plant species was confirmed through taxonomic authentication by a certified botanist, with corresponding voucher specimens at the herbarium of the Institute of Translational Medicine and Biotechnology, Sechenov University, Moscow, Russia.

The volatile composition of *Satureja montana* and *Origanum vulgare* hydrolates was evaluated using gas chromatography–mass spectrometry (GC–MS). Both samples revealed complex chemical profiles dominated by oxygenated monoterpenes, confirming their characteristic phytochemical patterns typical of the *Lamiaceae* family.

Despite their general similarity, the hydrolates differed in the ratio of key constituents as carvacrol, thymol, and p-cymene, which are known to contribute to antimicrobial and antioxidant activities. Minor variations in retention indices and relative abundances were observed, most likely due to differences in plant origin and hydrodistillation parameters. The results are summarized in Tables 1 and 2.

Table S1. Chemical composition of *Satureja montana* hydrolate

Compound	*Retention Index (RI)*	*Concentration (%)*
Carvacrol	1304	86.7
Thymol	1281	3.2
p-Cymene	1018	3.5
Linalool	1092	1.3
Borneol	1161	1.3
Terpinen-4-ol	1178	1.0
γ-Terpinene	1052	1.4
Myrcene	985	1.0
α-Terpineol	1189	0.7
1,8-Cineole	1030	0.5
Camphor	1143	tr

Notes: RI values were calculated relative to n-alkanes; concentrations are expressed as relative peak area percentages; tr – indicates trace amounts (<0.1%).

Table S2. Chemical composition of *Origanum vulgare* hydrolate

Compound	*Retention Index (RI)*	*Concentration (%)*
Carvacrol	1296	41.5
Thymol	1290	19.3
p-Cymene	1029	8.6
γ-Terpinene	1060	8.1
Linalool	1090	2.3
Sabinene	972	2.0
β-Myrcene	990	1.8
α-Pinene	938	1.0
Terpinen-4-ol	1172	1.2
α-Terpineol	1056	0.7
1,8-Cineole	982	0.5
Camphor	951	tr

Notes: RI values were calculated relative to n-alkanes; concentrations are expressed as relative peak area percentages; tr – indicates trace amounts (<0.1%).

Support material S2.

The basic diagram (Fig. 1) is taken with modifications from the source:

Efremov A.A. Exhaustive hydrodistillation method for obtaining essential oils from wild-growing plants // Advances in Current Natural Sciences. — 2013. — No. 7. — P. 88–94. — Available at: https://natural-sciences.ru/ru/article/view?id=32596 (accessed 26 September 2025).

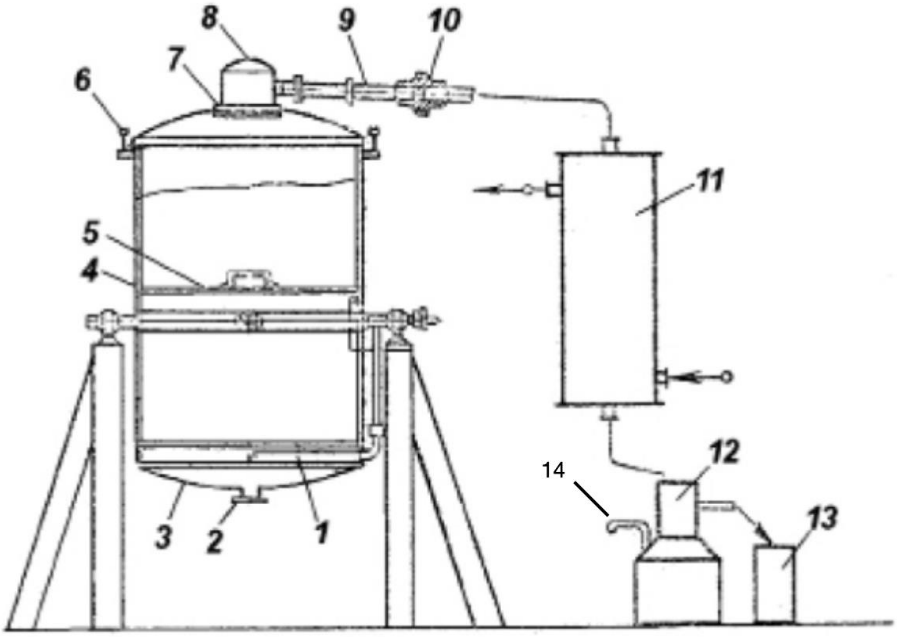

Figure S1. Distillation unit diagram (hydro-parodistillator): 1 – heating element; 2 – water drain valve; 3, 4 – steam jacket; 5 – partition; 6 – lid valve; 7 – distillation cube lid; 8, 9 – steam outlet pipe; 10 – steam outlet tube screw; 11 – condenser (refrigerator); 12 – receiver (Florentine flask); 13 – oil container; 14 – hydrolates.

Support material S3.

In essence, thermal injury transcends mere physical disruption; it represents a complex biological event that initiates a highly coordinated, yet frequently self-perpetuating, cascade of cellular dysfunction, microvascular compromise, and in-flammatory dysregulation.

Immediate pathological consequences of thermal exposure involve the development of distinct, concentric zones of tissue damage, a concept foundational to burn pathology, first described by Jackson in 1947 (Fig. 2) [1]. For example, the zone of coagulation, situated centrally, bears the brunt of the thermal energy, leading to irreversible protein denaturation, immediate cellular necrosis, and thrombotic occlusion of the microvascu-lature. Histologically, this region is characterized by profound cellular disorganization, nuclear pyknosis, karyorrhexis, and widespread coagulative necrosis affecting both epidermal and dermal components, rendering it non-viable and necessitating surgical debridement [2]. Surrounding this core is the zone of stasis, a critically important region marked by compromised microcirculation, endothelial dysfunction, and cellular ische-mia. While initially possessing some cellular viability, this zone is highly susceptible to secondary progression to irreversible necrosis if timely and effective resuscitation strat-egies are not implemented [3, 4]. Pathologically, this area exhibits endothelial cell swelling, increased leukocyte adhesion, and progressive

microthrombosis, all contrib-uting to a reduction in blood flow. The preservation of this potentially salvageable tissue remains a primary objective in acute burn management. Peripherally, the zone of hy-peremia represents the area of least severe injury, characterized by pronounced vaso-dilation and increased blood flow, reflecting an active inflammatory response. Cells within this zone are generally viable, and the injury is typically reversible, often re-solving spontaneously without significant intervention, though it contributes to local edema and pain perception [5].

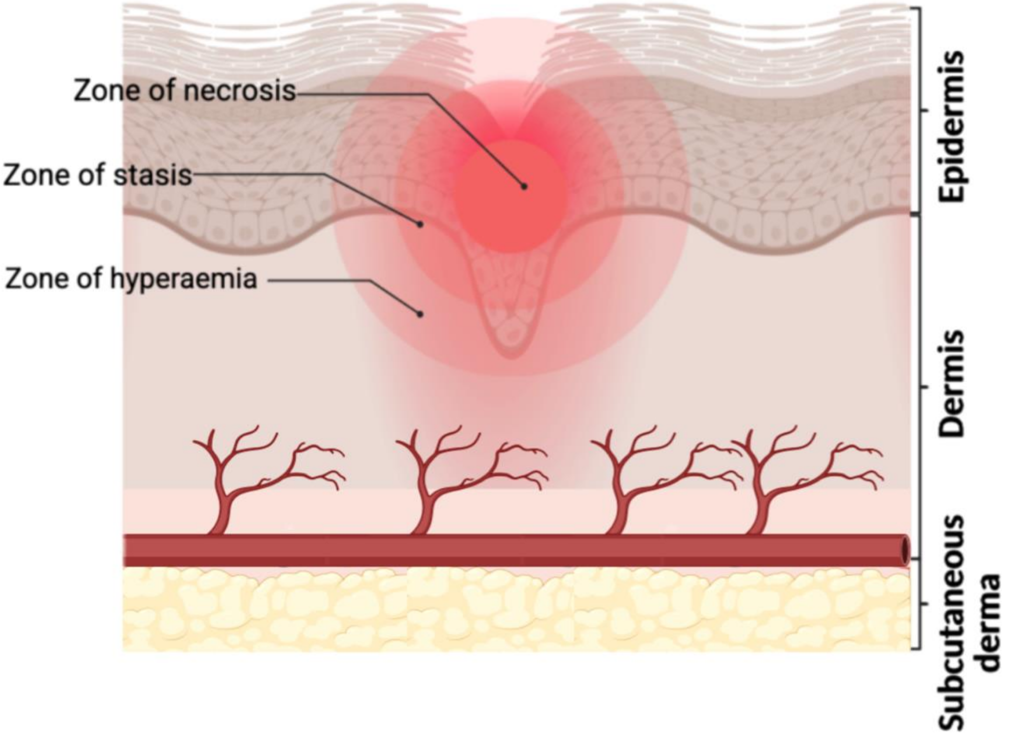

Figure S2. Schematic representation of Jackson's burn wound model, illustrating three concentric zones: the central zone of necrosis, the intermediate zone of stasis, and the peripheral zone of hy-peraemia.

References:

1. Hettiaratchy S, Dziewulski P. ABC of burns: pathophysiology and types of burns. BMJ. 2004 Jun 12;328(7453):1427-9. doi: 10.1136/bmj.328.7453.1427. Erratum in: BMJ. 2004 Jul 17;329(7458):148. PMID: 15191982; PMCID: PMC421790

2. Physio-pedia. Burn Wound Assessment. Available at: https://www.physio-pedia.com/Burn_Wound_Assessment (Accessed Sep 4, 2025)

3. Singer AJ, McClain SA, Taira BR, Guerriero JL, Zong W. Apoptosis and necrosis in the ischemic zone adjacent to third degree burns. Acad Emerg Med. 2008 Jun;15(6):549-54. doi: 10.1111/j.1553-2712.2008.00115.x. PMID: 18616442

4. Żwierełło W, Piorun K, Skórka-Majewicz M, Maruszewska A, Antoniewski J, Gutowska I. Burns: Classification, Pathophysiology, and Treatment: A Review. Int J Mol

Sci. 2023 Feb 13;24(4):3749. doi: 10.3390/ijms24043749. PMID: 36835171; PMCID: PMC9959609

5. Kaddoura I, Abu-Sittah G, Ibrahim A, Karamanoukian R, Papazian N. Burn injury: review of pathophysiology and therapeutic modalities in major burns. Ann Burns Fire Disasters. 2017 Jun 30;30(2):95-102. PMID: 29021720; PMCID: PMC5627559

Support material S4.

The colonization of burn wounds by *Staphylococcus aureus* represents a critical inflection point in the progression of thermal injury, moving the pathology from a sterile inflammatory process to a complex, infected wound state. This Gram-positive pathogen is uniquely equipped to thrive in the compromised burn microenvironment, primarily through its capacity for robust biofilm formation (Fig. 3). This extracellular matrix acts as a physical and chemical shield, protecting the bacterial community from both host immune defenses (e.g., phagocytosis) and conventional antimicrobial agents, thereby establishing a persistent, chronic infection focus [1].

Furthermore, the virulence of *S. aureus* is significantly potentiated by its extensive arsenal of secreted toxins. These include potent cytotoxins like alpha-hemolysin, panton–valentine leukocidin (PVL), exfoliative toxins A and B, toxic shock syndrome toxin-1, and other toxins [2]. These molecules actively disrupt host cell membranes, induce massive tissue necrosis, and trigger an exaggerated, often dysregulated, pro-inflammatory response. This toxin-mediated damage contributes to a spectrum of complications, ranging from localized wound deterioration to systemic sepsis and multi-organ failure [3]. The clinical urgency is compounded by the increasing prevalence of antibiotic-resistant strains, notably MRSA, which renders standard-of-care antimicrobial therapies ineffective. The necessity for targeted research to develop effective, multi-mechanistic strategies against this pathogen in the context of burn wounds is therefore paramount, directly informing the rationale for our study of *Lamiaceae* hydrolates.

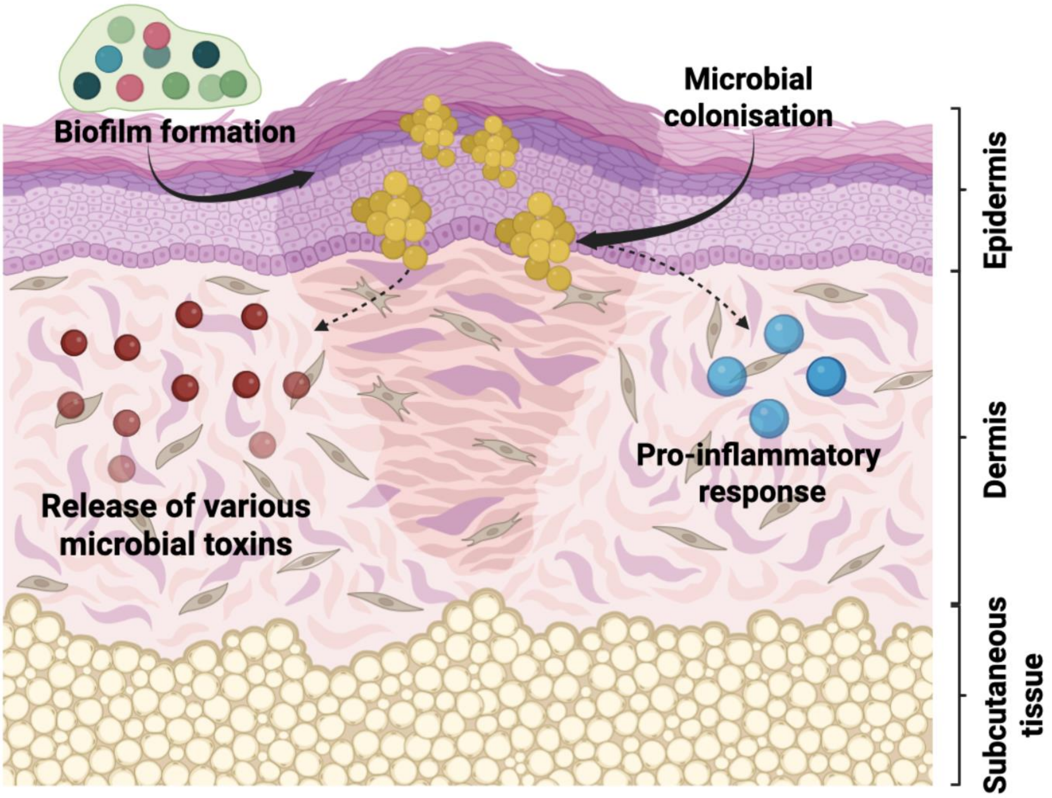

Figure S3. Schematic representation of *Staphylococcus aureus* infection in burn wounds, illustrating bacterial colonization, biofilm formation, and toxin-mediated immune activation.

References:

1. Peng Q, Tang X, Dong W, Sun N, Yuan W. A Review of Biofilm Formation of Staphylococcus aureus and Its Regulation Mechanism. Antibiotics (Basel). 2022 Dec 22;12(1):12. doi: 10.3390/antibiotics12010012. PMID: 36671212; PMCID: PMC9854888

2. Touaitia R, Mairi A, Ibrahim NA, Basher NS, Idres T, Touati A. Staphylococcus aureus: A Review of the Pathogenesis and Virulence Mechanisms. Antibiotics (Basel). 2025 May 6;14(5):470. doi: 10.3390/antibiotics14050470. PMID: 40426537; PMCID: PMC12108373

3. Salam MA, Al-Amin MY, Salam MT, Pawar JS, Akhter N, Rabaan AA, Alqumber MAA. Antimicrobial Resistance: A Growing Serious Threat for Global Public Health. Healthcare (Basel). 2023 Jul 5;11(13):1946. doi: 10.3390/healthcare11131946. PMID: 37444780; PMCID: PMC10340576
